# One More Effort

**Published:** 1890-11

**Authors:** 


### ONE MORE EFFORT.

Attention is called to the card from Dr. Cupples, Chairman Committee on Medical Legislation.

We cannot too strongly impress upon our readers and upon the medical profession throughout the State the necessity of prompt and energetic action in the matter referred to by Dr. Cupples. The legislature will meet in January, and by the 20th of December all these petitions should be in the hands of the committee, and each one should have five hundred signatures. This is the people's petition, not ours, and although a move of the highest political importance, it is not a "political scheme" as understood by the people. There is nothing selfish about it; the people and not the medical profession are the ones sought to be benefited by the movement; and when we say the people, it includes the fair portion of our population, and our friends and co-laborers should secure the signatures of prominent and influential women to the petitions as well as those of men. Women are usually foremost in all good work; invite them to take part in this. If anything, they are more interested in preventive medicine than any other class of the community; for, when sickness and death come, they are the sufferers doubly, the drudgery of nursing falls on them, and in the death of the male members of the community, who are the real sufferers? The women and children who are thus robbed of their support and protection. By all means get the women of Texas aroused to the necessity of preventing disease; and open their eyes also to that other danger—greater than disease—the danger of being legally murdered, by an incompetent, though registered, perhaps, diplomaed Doctor. Justice, every consideration of right demands that no one should be permitted to practice medicine but those who can show that they can intelligently do so; that they have been educated to the calling.

Our past failures in our efforts to secure a State Board of Health and a law to regulate the practice, should not discourage us. The season is now ripe for action—the people are aroused and our legislators know that it is not the Doctors who want and ask for protection—but the two and a half millons of industrious inhabitants of a great State, who realize that as yet they are only partially protected even from imported disease, and not at all from domestic or home made disease; while they are entirely at the mercy of the army of quacks and impostors who call themselves Doctors, and prey upon the credulity or ignorance of the people.

The JOURNAL wishes the committee God speed. But, before we call upon Hercules we must put our shoulders to the wheel. We have done so, and now, with one pull, a long pull, a strong pull, and above all, a pull all together, we hope to lift our beloved State out of the quag-mire of quackery, and place her on solid ground side by side with other enlightened States, which have long since seen the advantages and necessity of such legislation as we are asking for, and have provided boards of health, the "palladium" not of the "people's liberties," but of their safety, their health and their lives.

As to quarantine, maritime and frontier, the present system does well enough; but a quarantine law is not a board of health. We want—the JOURNAL'S preference is for—a board of health *built upon* the present system of quarantine—something else is needed besides quarantine. Let the State Health Officer administer quarantine unincumbered, but give him associaties to constitute, with himself, a full board which shall exercise all the functions of a board of health, not covered by the item of quarantine. But we are asking for a board, and must take what the State may give us.

